# Depressive Symptoms and Their Impact on Quality of Life in Parkinson’s Disease: An Exploratory Network Analysis Approach

**DOI:** 10.3390/jcm12144616

**Published:** 2023-07-11

**Authors:** Konstantin G. Heimrich, Sarah Mendorf, Aline Schönenberg, Diego Santos-García, Pablo Mir, COPPADIS Study Group, Tino Prell

**Affiliations:** 1 Department of Neurology, Jena University Hospital, Am Klinikum 1, 07747 Jena, Germany; 2 Department of Geriatrics, Halle University Hospital, Ernst-Grube-Straße 40, 06120 Halle, Germany; 3 Department of Neurology, CHUAC (Complejo Hospitalario Universitario de A Coruña), c/As Xubias 84, 15006 A Coruña, Spain; 4 Unidad de Trastornos del Movimiento, Servicio de Neurología y Neurofisiología Clínica, Instituto de Biomedicina de Sevilla, Hospital Universitario Virgen del Rocío/CSIC/Universidad de Sevilla, 41013 Seville, Spain; 5 Centro de Investigación Biomédica en Red Sobre Enfermedades Neurodegenerativas (CIBERNED), 28031 Madrid, Spain; 6 Fundación Española de Ayuda a la Investigación en Enfermedades Neurodegenerativas y/o de Origen Genético, Calle Antonio J de Sucre 1A, 15179 Oleiros, Spain

**Keywords:** Parkinson’s disease, quality of life, BDI-II, depression, fatigue, network analysis

## Abstract

The clinical presentation of Parkinson’s disease (PD) is often dominated by depressive symptoms, which can significantly impact the patients’ quality of life (QoL). However, it is not clear how these depressive symptoms are interconnected, or if some symptoms are more influential in affecting QoL. In the Cohort of Patients with Parkinson’s Disease in Spain (COPPADIS) study, 686 patients with PD were analyzed using network analyses. The patients completed the Beck Depression Inventory II (BDI-II) and provided their overall QoL (EUROHIS-QOL) at the beginning of the study. The study used centrality measures such as Expected Influence and Bridge Expected Influence to identify depressive symptoms that had the greatest impact on overall QoL. The results of exploratory network analyses indicate that the BDI-II items related to *loss of energy*, *past failure*, and *tiredness or fatigue* have the greatest impact on overall QoL as measured by the EUROHIS-QOL 8-item index. The *loss of energy* and *tiredness or fatigue* BDI-II items are also strongly associated with a number of different EUROHIS-QOL items, according to Bridge Expected Influences. For individuals suffering from PD, network analysis can aid in identifying significant non-motor symptoms that impact their QoL, thus paving the way for potential improvements.

## 1. Introduction

Parkinson’s disease (PD) is one of the most common progressive neurodegenerative disorders and characterized by motor and various non-motor symptoms [1]. Depression is a crucial factor that determines the quality of life (QoL) in individuals with PD, and it is a particularly significant non-motor symptom [2,3,4,5]. The overall QoL and health-related QoL are critical outcomes of healthcare, and they are important predictors of morbidity and mortality [6,7]. Therefore, identifying and treating depression is essential to maintain QoL in individuals with PD. However, just a small number of individuals with PD who report depressive symptoms fulfill the diagnostic criteria for a major depressive disorder as defined by the Diagnostic and Statistical Manual of Mental Disorders (DSM) [8,9]. Nonetheless, even subthreshold depressive symptoms can negatively affect the QoL of individuals with PD [10]. To provide tailored treatment for individuals with PD and depressive symptoms, it is important to determine which depressive symptoms have the greatest impact on general QoL and different domains of QoL.

To address this inquiry, one must confront various methodological challenges. Measuring and diagnosing depression in PD presents difficulties since there are multiple distinct measures available. Diagnosis and thus prevalence may vary depending on the definition of depressive disorders, e.g., *major depression* according to DSM criteria or *depressive episode* according to the International Classification of Diseases (ICD), as well as various self-report or clinician-rated psychiatric symptom rating scales [9,11]. In addition, there is significant overlap between depression and other non-motor symptoms of PD [7,12,13]. Additionally, different depressive symptoms may have the same or opposite effect on QoL, making it challenging to determine the one-directional effect of these symptoms. Consequently, conventional statistical methods may not capture the intricate interplay between depressive symptoms and QoL measures. This presents an exciting scientific challenge that requires careful consideration.

The method of network analysis is a promising way to model interactions between a large number of variables, which is particularly important for the study of mental health problems [14]. Unlike classical regression modeling, which reduces the structure of variables to their shared information, network analysis directly estimates the associations between all variables [15,16]. This exploratory approach allows for the visualization of the relationships between multiple variables without the assumption of a direction of effects. In this study, we implemented network analysis to reveal the complex interactive relationship between depressive symptoms and QoL in people with PD (PwPD).

## 2. Materials and Methods

### 2.1. Study Design and Participants

We used data from the national, multicenter, and longitudinal Cohort of Patients with Parkinson’s Disease in Spain (COPPADIS) study [17]. Non-demented PwPD between 30 and 75 years were recruited from January 2016 to November 2017. More detailed information regarding the study design, content, and exclusion criteria can be found in the COPPADIS study protocol [17]. We selected PwPD for whom measures of depression and QoL were available at baseline (N = 686).

### 2.2. Extracted Variables

For measurement of depressive symptoms, the Beck Depression Inventory II (BDI-II) was used. The BDI-II consists of 21 items (each rated on a 4-point Likert scale ranging from zero to three). The summed total score ranges from 0 to 63 points, with higher values indicating more depressive symptoms [18]. In our network analysis, we refrained from classifying patients into two categories of having or not having depression. We felt that even the presence of subsyndromal depression or some depressive symptoms could impact the QoL. However, for descriptive statistics, we relied on the established BDI threshold to identify patients with depression.

QoL was assessed with the EUROHIS-QOL [19]. The EUROHIS-QOL was derived from the World Health Organization Quality of Life assessment [20]. It consists of eight items given on a 5-point Likert scale and covers psychological, physical, social, and environmental domains of QoL [19]: How would you rate your quality of life? (*QOL*); How satisfied are you with your health? (*HEA*); Do you have enough energy for everyday life? (*ENE*); How satisfied are you with your ability to perform your daily living activities? (*ACT*); How satisfied are you with yourself? (*YOU*); How satisfied are you with your personal relationships? (*REL*); Have you enough money to meet your needs? (*MON*); How satisfied are you with the conditions of your living place? (*LIV*).

An index of overall QoL (EUROHIS-QOL 8-item index) is calculated by summation of the scores of every item, whereby higher values indicate a better QoL [21].

Furthermore, the following variables were obtained to describe the cohort: age, sex, Hoehn and Yahr stage [22], Unified Parkinson’s Disease Rating Scale (UPDRS) parts III and IV [23], total scores of the Non-Motor Symptoms Scale in Parkinson’s disease (NMSS) [24], and the Mini-Mental State Examination (MMSE) [25].

### 2.3. Statistical Analyses

For statistical analyses, we used R (version 4.2.1, R Foundation for Statistical Computing, Vienna, Austria), SPSS (IBM SPSS Statistics 27, IBM, Armonk, NY, USA), and JASP (version 0.15, JASP Team, Amsterdam, The Netherlands). For the characterization of the cohort, descriptive statistics were applied. Data were tested for normality by using the Shapiro–Wilk test. For non-normally distributed data, the median and interquartile range were determined. The level of statistical significance for all tests was set at *p* < 0.05 (two-tailed).

Exploratory network analyses were conducted to explore the associations between the 21 BDI-II items and the EUROHIS-QOL. In a network, the whole complex interacting system between various symptoms is used to understand their connections. A regularization technique was used to prevent overfitting the structure of the network [26], called the extended Bayesian information criterion (EBIC) [27,28] with the least absolute shrinkage and selection operator (LASSO) [29]. Due to the ordinal structure of the data, polychoric correlations were estimated [30]. The tuning parameter of EBICglasso was set to 0.5 to allow more sensitive and specific network analysis. The items of the questionnaires are displayed by the nodes of the network and positioned by the Fruchterman–Reingold algorithm [31]. The connections between nodes are called edges and their thickness indicates the strength of the correlations. Correlations were classified as low (|r|= 0.1), moderate (|r| = 0.3), or strong (|r| = 0.5) [32]. Blue edges refer to a positive correlation, and red edges to a negative one. 

To describe the network, the centrality measure *Expected Influence* was determined by relative values. The Expected Influence of a node is defined as the sum of the absolute edge weights that are connected to that node, taking into account positive and negative edges [33]. Additionally, the centrality measure *Bridge Expected Influence* (1-step) was determined using relative values. Bridge Expected Influence refers to the sum of the value of all edges that exist between a node of one community (i.e., a depressive symptom) and all nodes of another community (i.e., the eight items of the EUROHIS-QOL) [34]. 

Moreover, we determined nodewise predictability to determine how well a given node of a network (i.e., the EUROHIS-QOL 8-item index) is predictable by all nodes directly connected to it (i.e., associated depressive symptoms) [35]. The obtained explained variance R^2^ can be between 0 and 1, and values ≥0.13 are defined as moderate and ≥0.26 as high [32]. 

Network stability was assessed by means of the correlation stability (CS) coefficient (number of case-dropping bootstraps = 1000). The CS coefficient quantifies the proportion of cases which can be omitted to still maintain a correlation with the original centrality measure that is at minimum 0.7 in at least 95% of samples [16]. The CS coefficient should be in general above 0.25 and preferable above 0.5 [16]. 

## 3. Results

### 3.1. Descriptive Analyses

Descriptive statistics of the study population are shown in Table 1. Of the 686 PwPD, 274 (39.9%) were female and 412 (60.1%) were male. Patients had a median age of 64 years (IQR = 57–70 years), and a median duration of the disease of five years (IQR = 2–8 years). Most patients had a disease stage with bilateral involvement (Hoehn and Yahr stage ≥ 2), and moderate motor impairment (median UPDRS III: 21 points; IQR = 14–30). Frequently, they experienced one motor complication (median UPDRS IV: 1 point; IQR = 0–3). Patients presented non-motor symptoms according to the NMSS with a median total score of 35 points (IQR = 19–61). The median BDI-II total score was 7 points (IQR = 3–13). Using the previously described BDI-II cut-offs, 16.2% (N = 111) had depression, 27.0% (N = 185) had subthreshold depression, and 56.9% (N = 390) had no depression [36]. 

### 3.2. Network Analyses

To explore the links between depressive symptoms (21 BDI-II items) and EUROHIS-QOL, network analyses were conducted. The study examined two models: the first model included the EUROHIS-QOL 8-item index, while the second model included all eight items of the EUROHIS-QOL.

#### 3.2.1. Network Model 1: Association between BDI-II Items and Overall QoL (EUROHIS-QOL 8-Item Index)

**Figure 1:**
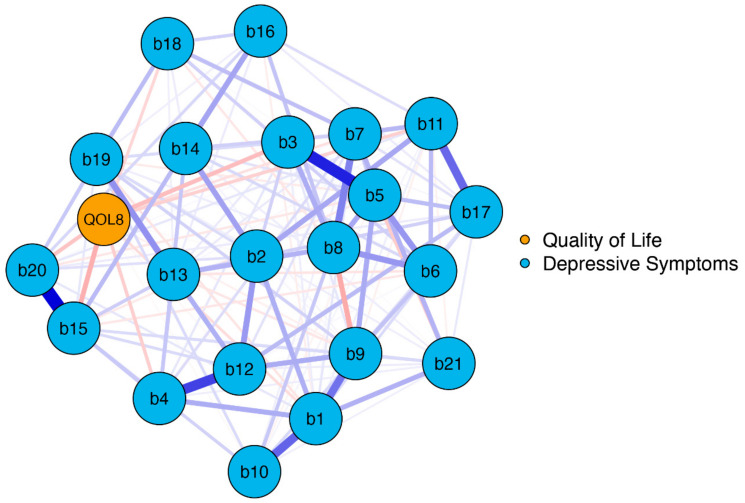
Network structure BDI-II items and EUROHIS-QOL 8-item index (*model 1*). The blue nodes display the items of the BDI-II (*b1–b21*), and the orange node displays the summed EUROHIS-QOL 8-item index (*QOL8*). The edges display the correlations between the nodes. Blue edges represent positive associations, and red edges represent negative associations. The thickness of the edges indicate how strong these connections are. BDI-II: revised Beck Depression Inventory (b1: Sadness; b2: Pessimism; b3: Past failure; b4: Loss of pleasure; b5: Guilty feelings; b6: Punishment feelings; b7: Self-dislike; b8: Self-criticalness; b9: Suicidal thoughts or wishes; b10: Crying; b11: Agitation; b12: Loss of interest; b13: Indecisiveness; b14: Worthlessness; b15: Loss of energy; b16: Changes in sleeping pattern; b17: Irritability; b18: Changes in appetite; b19: Concentration difficulty; b20: Tiredness or fatigue; b21: Loss of interest in sex); EUROHIS-QOL: European Union Health Interview Survey for Quality Of Life.

The network plot of *model 1* is shown in Figure 1. The blue nodes display the items of the BDI-II (*b1–b21*), and the orange node displays the EUROHIS-QOL 8-item index (*QOL8*) as a measure of overall QoL. 

On a global level, the network was well-connected (137 of 231 non-zero edges) without isolated nodes. The BDI-II and QOL8 nodes had numerous interactions, primarily negative ones (indicated by red edges). This means that depressive symptoms were linked to poorer QoL. The strongest negative connections were observed between *QOL8*–*loss of energy* (*b15*), *QOL8*–*past failure* (b3), and *QOL8*–*tiredness or fatigue* (b20), with edge weights detailed in Table S1.

**Figure 2:**
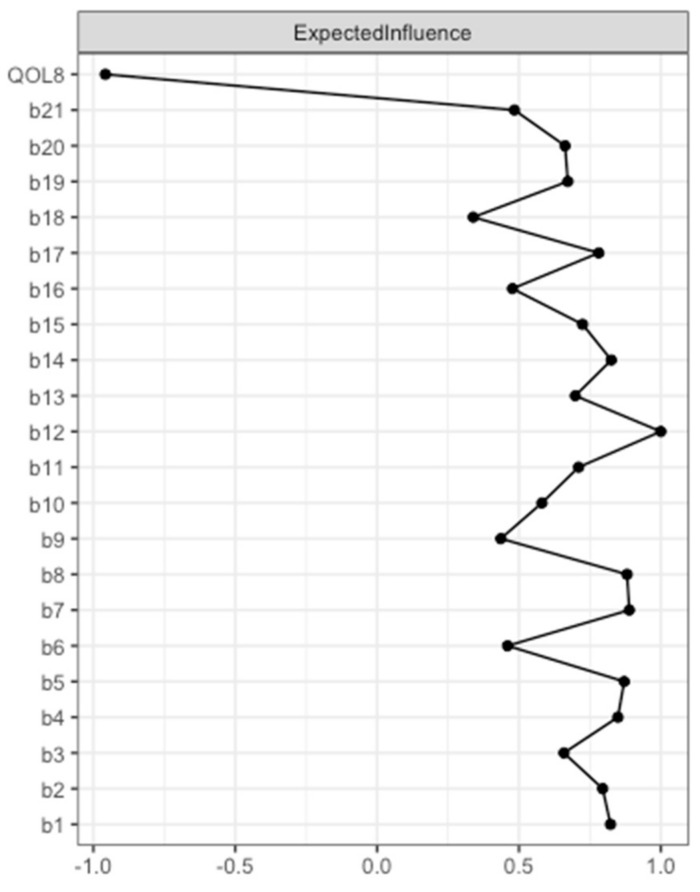
Expected Influence BDI-II items and EUROHIS-QOL 8-item index (*model 1*). Expected Influence centrality measures of the items of the BDI-II (*b1–b21*) and the EUROHIS-QOL 8-item index (*QOL8*) are given in relative values. BDI-II: revised Beck Depression Inventory (b1: Sadness; b2: Pessimism; b3: Past failure; b4: Loss of pleasure; b5: Guilty feelings; b6: Punishment feelings; b7: Self-dislike; b8: Self-criticalness; b9: Suicidal thoughts or wishes; b10: Crying; b11: Agitation; b12: Loss of interest; b13: Indecisiveness; b14: Worthlessness; b15: Loss of energy; b16: Changes in sleeping pattern; b17: Irritability; b18: Changes in appetite; b19: Concentration difficulty; b20: Tiredness or fatigue; b21: Loss of interest in sex); EUROHIS-QOL: European Union Health Interview Survey for Quality Of Life.

Expected Influence was determined for each node (see Figure 2, and Table S1). A high Expected Influence means that changing the value of this node can have a rapid effect on other nodes within the network. Here, *QOL8* had the strongest negative Expected Influence. Accordingly, *QOL8* had the highest negative input weights from other nodes that are directly connected. The 13 nodes that were directly connected to *QOL8 (sadness*, *pessimism*, *past failure*, *loss of pleasure*, *self-dislike*, *suicidal thoughts or wishes*, *crying*, *agitation*, *loss of interest*, *indecisiveness*, *loss of energy*, *changes in appetite*, *tiredness or fatigue)* explained 42.8% of *QOL8* variance, as revealed by nodewise predictability analysis (see Table S1).

According to the case-dropping bootstrapped procedure, the network can be considered stable as the CS coefficient of Expected Influence remained high (CS(cor = 0.7) = 0.67) (see Figure S1). 

#### 3.2.2. Network Model 2: Association between BDI-II Items and the Eight EUROHIS-QOL Items

**Figure 3:**
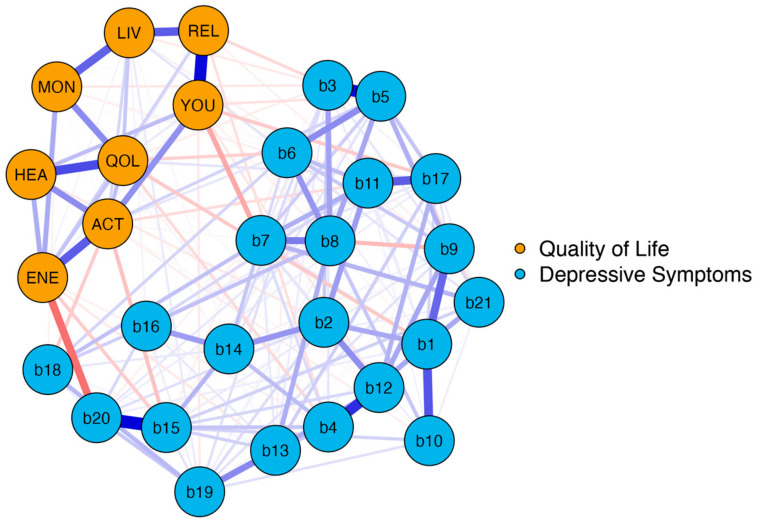
Network structure BDI-II items and EUROHIS-QOL items (*model 2*). The blue nodes display the items of the BDI-II (*b1–b21*), and the orange nodes display the eight items of the EUROHIS-QOL. The edges display the correlations between the nodes., Blue edges represent positive associations, and red edges represent negative associations. The thickness of the edges indicate how strong these connections are. BDI-II: revised Beck Depression Inventory (b1: Sadness; b2: Pessimism; b3: Past failure; b4: Loss of pleasure; b5: Guilty feelings; b6: Punishment feelings; b7: Self-dislike; b8: Self-criticalness; b9: Suicidal thoughts or wishes; b10: Crying; b11: Agitation; b12: Loss of interest; b13: Indecisiveness; b14: Worthlessness; b15: Loss of energy; b16: Changes in sleeping pattern; b17: Irritability; b18: Changes in appetite; b19: Concentration difficulty; b20: Tiredness or fatigue; b21: Loss of interest in sex); EUROHIS-QOL: European Union Health Interview Survey for Quality Of Life (QOL: How would you rate your quality of life?; HEA: How satisfied are you with your health?; ENE: Do you have enough energy for everyday life?; ACT: How satisfied are you with your ability to perform your daily living activities?; YOU: How satisfied are you with yourself?; REL: How satisfied are you with your personal relationships?; MON: Have you enough money to meet your needs?; LIV: How satisfied are you with the conditions of your living place?).

In the second network, we analyzed the association between BDI-II items and the distinct EUROHIS-QOL items. The network plot of *model 2* is shown in Figure 3. The blue nodes represent the BDI-II items (*b1–b21*) and the orange nodes represent the eight items of the EUROHIS-QOL. 

**Figure 4:**
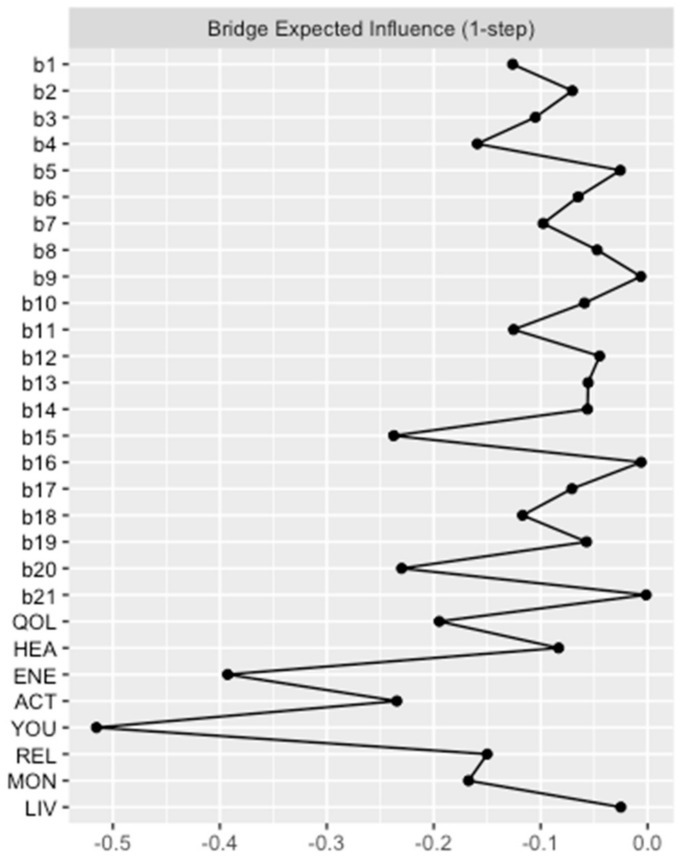
Bridge Expected Influence BDI-II items and EUROHIS-QOL items (*model 2*). Bridge Expected Influence centrality measures of the items of the BDI-II (*b1–b21*) and the eight items of the EUROHIS-QOL are given in relative values. BDI-II: revised Beck Depression Inventory (b1: Sadness; b2: Pessimism; b3: Past failure; b4: Loss of pleasure; b5: Guilty feelings; b6: Punishment feelings; b7: Self-dislike; b8: Self-criticalness; b9: Suicidal thoughts or wishes; b10: Crying; b11: Agitation; b12: Loss of interest; b13: Indecisiveness; b14: Worthlessness; b15: Loss of energy; b16: Changes in sleeping pattern; b17: Irritability; b18: Changes in appetite; b19: Concentration difficulty; b20: Tiredness or fatigue; b21: Loss of interest in sex); EUROHIS-QOL: European Union Health Interview Survey for Quality Of Life (QOL: How would you rate your quality of life?; HEA: How satisfied are you with your health?; ENE: Do you have enough energy for everyday life?; ACT: How satisfied are you with your ability to perform your daily living activities?; YOU: How satisfied are you with yourself?; REL: How satisfied are you with your personal relationships?; MON: Have you enough money to meet your needs?; LIV: How satisfied are you with the conditions of your living place?).

Again, network analysis demonstrated a well-connected network (183 of 406 non-zero edges) without isolated nodes. One can distinguish two parts reflecting the two different questionnaires. However, there were many interactions between the nodes of the BDI-II and the EUROHIS-QOL. These interactions were primarily negative, as depicted by the red edges. This implies that a lower intensity of depressive symptoms is associated with a higher QoL. The strongest negative connection between the two parts was observed between “tiredness or fatigue” and “Do you have enough energy for everyday life?” (*b20* and *ENE*), which is not surprising as both are similar constructs. However, identifying the depressive symptoms that are linked with all eight items on the EUROHIS-QOL questionnaire is a matter of great interest. This is why Bridge Expected Influences were determined. The Bridge Expected Influence centrality measures are shown in Figure 4 (and also tabulated in Table S2). The nodes *b15* (loss of energy) and *b20* (tiredness or fatigue) were found to have the highest Bridge Expected Influences, suggesting that they are the most strongly associated depressive symptoms with all eight items on the EUROHIS-QOL questionnaire. Thereby, node *b15* is associated in particular with *ACT* (How satisfied are you with your ability to perform your daily living activities?) (edge weight: −0.102). This means that feeling loss of energy has greatest influence on the ability to perform daily living activities among the QoL domains. Moreover, node *b20* showed an association with *ENE* (Do you have enough energy for everyday life?) of the quality-of-life domain (edge weight: −0.236). Respectively, patients who reported tiredness or fatigue had less energy for everyday life. As revealed by the network plot of *model 2* (Figure 3), these two depressive symptoms with the highest Bridge Expected Influence (*loss of energy* and *tiredness or fatigue*) were closely connected to each other. *Loss of pleasure* (*b4*) was the third influential depressive symptom, which is especially associated with *QOL* (How would you rate your quality of life?; edge weight: −0.063) and *REL* (How satisfied are you with your personal relationships?; edge weight: −0.053).

According to the case-dropping bootstrapped procedure, the network can be considered stable as the CS coefficient of Bridge Expected Influence remained high (CS(cor = 0.7) = 0.60) (see Figure S2).

## 4. Discussion

Our research utilized network analysis to uncover intricate relationships between depressive symptoms and QoL in PwPD. Our findings confirmed that there is a strong correlation between the 21 items of the BDI-II and EUROHIS-QOL, whether we considered the EUROHIS-QOL 8-item index (model 1) or all eight items together (model 2). These correlations are largely negative, indicating that lower levels of depressive symptoms are associated with higher QoL. Specifically, the two BDI-II items related to *loss of energy* (b15) and *tiredness or fatigue* (b20) had the most significant impact on QoL. Furthermore, our network analysis revealed that these two BDI-II items (b15 and b20) were closely linked to each other.

When evaluating the impact of depressive symptoms on QoL using the EUROHIS-QOL 8-item index, our network analysis (model 1) found that feelings of *loss of energy* (b15), *past failure* (b3), and *tiredness or fatigue* (b20) were influential symptoms. Considering the impact of depressive symptoms on all eight items of the EUROHIS-QOL (model 2), network analysis revealed negative associations between QoL and especially *loss of energy* (b15) and *tiredness or fatigue* (b20). Taking into account the complex interactions of all 21 BDI-II items and the eight QoL measures, network analysis demonstrated that both symptoms are closely connected to each other. In addition, *loss of pleasure* (b4) was identified as the third influential depressive symptom as revealed by Bridge Expected Influence.

In summary, the most influential depressive symptoms affecting both the EUROHIS-QOL 8-item index and all eight EUROHIS-QOL items together were *loss of energy* (b15) and feelings of *tiredness or fatigue* (b20). Overall, this study sheds light on the importance of considering the complex interplay between depressive symptoms and QoL measures.

Fatigue is generally described as an all-encompassing feeling of tiredness, decreased energy, and often a sense of complete depletion. It should not be confused with symptoms of depression, such as feelings of worthlessness, despair, or hopelessness, although it can be a sign of depression. Additionally, it is not equivalent to limb weakness or any visible sign of physical weakness [37]. The definition indicates that loss of energy may be part of the clinical picture of fatigue. However, these symptoms are considered separate components of the BDI-II since not all individuals with loss of energy experience established fatigue. Nevertheless, our research has revealed that both aspects are closely linked to one another.

One of the most prevalent and disabling non-motor symptoms in PwPD is fatigue [38], which can manifest even in the early stages of the disease [39] and often persists or worsens over time [40,41]. This can lead to decreased participation in social and recreational activities [42,43], negatively impacting the patients’ QoL [38,44]. This was also demonstrated in this study, in which both BDI-II items (*loss of energy* and *tiredness or fatigue*) had a strong influence on QoL. Therefore, identification and treatment of fatigue in PD appears to be promising to improve patients’ QoL. However, there are currently no specific guidelines for managing PD-related fatigue, and research has not provided enough evidence to support the use of pharmaceutical or non-pharmaceutical treatments [45,46]. The primary and essential step in managing fatigue in PD is to educate patients and their families about its common occurrence.

Another BDI-II item that has great influence on QoL was *past failure* (b3). The experience of past failures can affect QoL [47,48], leading to negative psychological outcomes [49,50]. Individuals who have experienced setbacks, failures, or negative life events such as divorce or job loss, may attribute blame to themselves and experience shame due to their inability to meet their own or others’ expectations in various aspects of life [51,52]. These feelings of shame can also contribute to a lower QoL and increased susceptibility to psychopathological symptoms [53,54].

Moreover, the presence of *loss of pleasure* (b4), also known as anhedonia, is of central importance within the network. Anhedonia is seen as the inability to feel pleasure from activities or experiences that are normally enjoyable or rewarding [55]. Therefore, anhedonia can have a significant impact on QoL [56,57]. 

Using network analyses, our previous study revealed the impact of depressive symptoms in PwPD [58,59]. It was demonstrated that in particular feelings of sadness are important within the complex interacting network of non-motor symptoms in PD, as assessed by the NMSS [58]. Furthermore, fatigue was identified as non-motor symptom that is most strongly associated with health-related QoL, as assessed by the Parkinson’s Disease Questionnaire 39 (PDQ-39) summary index and in particular the mobility and activities of daily living subscales of the PDQ-39 [59]. These findings demonstrate that depression is a heterogenous construct, and depressive symptoms often overlap with other non-motor symptoms in PD [60].

Our study has shown that the loss of energy and constant fatigue are crucial factors in the network of depressive symptoms and QoL measures. These symptoms are categorized under the somatic factor of depression, as per the BDI-II [60]. However, *past failure* and *loss of pleasure* are also significant and fall under the affective factor of depression [60]. Overall, our findings shed light on the importance of these symptoms in understanding depression and its impact on an individual’s QoL.

Our research has shed light on the intricate interplay between symptoms of depression and QoL in PwPD. However, our study has certain limitations. The inclusion and exclusion criteria, such as age limit, absence of dementia or severe comorbidities, and lack of second-line therapies, mean that the data generated may not be entirely representative of the PD population. As a result, our findings cannot be applied to individuals with advanced cognitive decline or to those living in different countries with varying healthcare systems. Second, depressive symptoms and QoL measures were recorded on self-reports, that can depend on mood and motivation. In this context, fluctuations of depressive symptoms and QoL were not assessed. Third, in order to consider the largest study population we used baseline data from the COPPADIS study, which were obtained in 2016 and 2017. In general, a very large cohort is needed to perform a network analysis. A smaller cohort would significantly reduce the stability of the network. Accordingly, the dataset is about seven years old, which limits its comparability with more recent data. Fourth, network analysis is still an exploratory approach. Therefore, our data cannot describe causality between different symptoms or their effects on QoL longitudinally. Accordingly, further research is required to determine if influencing the most influential depressive symptoms can improve QoL.

## 5. Conclusions

Our study revealed complex interactions between depressive symptoms and QoL in PwPD. Especially *loss of energy* and feelings of *tiredness or fatigue* were identified as the most influential depressive symptoms. Furthermore, symptoms of *past failure* and *loss of pleasure* also appear to have a noteworthy impact. Further research is required to determine if influencing these symptoms can effectively improve QoL in PwPD. 

## Figures and Tables

**Table 1:** Descriptive statistics (N = 686).

Variable	
Age	64 (57–70)
Disease duration	5 (2–8)
HY off	2 (2–2)
UPDRS III off	21 (14–30)
UPDRS IV off	1 (0–3)
NMSS, total score	35 (19–61)
MMSE, total score	30 (29–30)
BDI-II, total score	7 (3–13)
1. Sadness	0 (0–1)
2. Pessimism	0 (0–1)
3. Past failure	0 (0–0)
4. Loss of pleasure	0 (0–1)
5. Guilty feelings	0 (0–0)
6. Punishment feelings	0 (0–0)
7. Self-dislike	0 (0–0)
8. Self-criticalness	0 (0–1)
9. Suicidal thoughts or wishes	0 (0–0)
10. Crying	0 (0–1)
11. Agitation	0 (0–1)
12. Loss of interest	0 (0–1)
13. Indecisiveness	0 (0–1)
14. Worthlessness	0 (0–1)
15. Loss of energy	1 (0–1)
16. Changes in sleeping pattern	1 (0–2)
17. Irritability	0 (0–1)
18. Changes in appetite	0 (0–1)
19. Concentration difficulty	1 (0–1)
20. Tiredness or fatigue	1 (0–1)
21. Loss of interest in sex	0 (0–1)
EUROHIS-QOL 8-item index	31 (28–33)
1. QOL, quality	4 (3–4)
2. HEA, health	3 (3–4)
3. ENE, energy	4 (3–4)
4. ACT, activities	4 (3–4)
5. YOU, yourself	4 (3–4)
6. REL, relationships	4 (4–4)
7. MON, money	4 (3–4)
8. LIV, living	4 (4–5)

Values are given as the medians and interquartile ranges. BDI-II: revised Beck Depression Inventory; EUROHIS-QOL: European Union Health Interview Survey for Quality Of Life (QOL: How would you rate your quality of life?; HEA: How satisfied are you with your health?; ENE: Do you have enough energy for everyday life?; ACT: How satisfied are you with your ability to perform your daily living activities?; YOU: How satisfied are you with yourself?; REL: How satisfied are you with your personal relationships?; MON: Have you enough money to meet your needs?; LIV: How satisfied are you with the conditions of your living place?); HY: Hoehn and Yahr stage; MMSE: Mini-Mental State Examination; N: number of participants; NMSS: Non-Motor Symptoms Scale in Parkinson’s Disease; UPDRS: Unified Parkinson’s Disease Rating Scale.

## Data Availability

The data presented in this study are available on request from D.S.-G. on behalf of the COPPADIS Study Group.

## Supplementary Materials

The following supporting information can be downloaded at: https://www.mdpi.com/article/10.3390/jcm12144616/s1, Figure S1: Case-dropping bootstrapped procedure of Expected Influence (*model 1)*; Figure S2: Case-dropping bootstrapped procedure of Bridge Expected Influence (*model 2*); Table S1: Network analysis BDI-II items and EUROHIS-QOL 8-item index; Table S2: Network analysis BDI-II items and EUROHIS-QOL items; Table S3: Affiliations of the collaborators of the COPPADIS Study Group.

## Appendix A

The Collaborators of the COPPADIS Study Group (affiliations are shown in Table S3): Adarmes AD, Almeria M, Alonso Losada MG, Alonso Cánovas A, Alonso Frech F, Alonso Redondo R, Álvarez I, Álvarez Sauco M, Aneiros Díaz A, Arnáiz S, Arribas S, Ascunce Vidondo A, Aguilar M, Ávila MA, Bernardo Lambrich N, Bejr-Kasem H, Blázquez Estrada M, Botí M, Borrue C, Buongiorno MT, Cabello González C, Cabo López I, Caballol N, Cámara Lorenzo A, Canfield Medina H, Carabajal Pendón E, Carrillo F, Carrillo Padilla FJ, Casas E, Catalán MJ, Clavero P, Cortina Fernández A, Cosgaya M, Cots Foraster A, Crespo Cuevas A, Cubo E, de Deus Fonticoba T, de Fábregues-Boixar O, Díez-Fairen M, Dotor García-Soto J, Erro E, Escalante S, Estelrich Peyret E, Fernández Guillán N, Gámez P, Gallego M, García Caldentey J, García Campos C, García Díez C, García Moreno JM, Gastón I, Gómez Garre MP, Gómez Mayordomo V, González Aloy J, González-Aramburu I, González Ardura J, González García B, González Palmás MJ, González Toledo GR, Golpe Díaz A, Grau Solá M, Guardia G, Hernández Vara J, Horta-Barba A, Idoate Calderón D, Infante J, Jesús S, Kulisevsky J, Kurtis M, Labandeira C, Labrador MA, Lacruz F, Lage Castro M, Lastres Gómez S, Legarda I, López Ariztegui N, López Díaz LM, López Domínguez D, López Manzanares L, López Seoane B, Lucas del Pozo S, Macías Y, Mata M, Martí Andres G, Martí MJ, Martínez Castrillo JC, Martinez-Martin P, McAfee D, Meitín MT, Mendoza Plasencia Z, Menéndez González M, Méndez del Barrio C, Mir P, Miranda Santiago J, Morales Casado MI, Moreno Diéguez A, Muro García I, Nogueira V, Novo Amado A, Novo Ponte S, Ordás C, Pagonabarraga J, Pareés I, Pascual-Sedano B, Pastor P, Pérez Fuertes A, Pérez Noguera R, Planas-Ballvé A, Planellas L, Prats MA, Prieto Jurczynska C, Puente V, Pueyo Morlans M, Puig Daví A, Redondo Rafales N, Rodríguez Méndez L, Rodríguez Pérez AB, Roldán F, Ruíz De Arcos M, Ruíz Martínez J, Sánchez Alonso P, Sánchez-Carpintero M, Sánchez Díez G, Sánchez Rodríguez A, Santacruz P, Santos García D, Segundo Rodríguez JC, Seijo M, Sierra Peña M, Solano Vila B, Suárez Castro E, Tartari JP, Valero C, Vargas L, Vela L, Villanueva C, Vives B.
