# Supplementary material for: Depressive Symptoms and Their Impact on Quality of Life in Parkinson’s Disease: An Exploratory Network Analysis Approach
Source: J Clin Med. 2023 Jul 11;12(14):4616. doi: 10.3390/jcm12144616 (PMC10380984; doi:10.3390/jcm12144616)
Supplement: Supplementary file 1 [file jcm-12-04616-s001.zip › jcm_nwbdi_supp_1.0.docx]

**
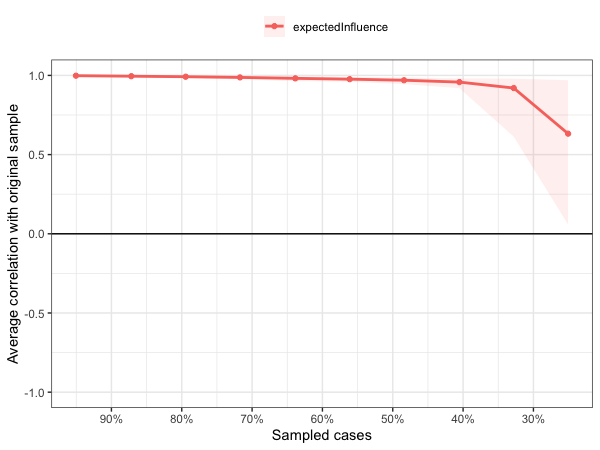
**

**Figure S1:** Case-dropping bootstrapped procedure of Expected Influence (*model 1)*(number of bootstraps = 1000). The correlations of the centrality measure Expected Influence between the original sample and those from the subsamples with an increasingly higher percentage of dropout cases were calculated. The correlation stability coefficient (CS coefficient) represents the maximum proportion of cases that can be dropped to retain a correlation of at least 0.70 with the original Expected Influence in at least 95% of the samples. The 95% confidence interval of the correlation is indicated. The case-dropping bootstrap procedure showed that CS coefficient of node Expected Influence (CS(cor=0.7) = 0.67) remained high.

**
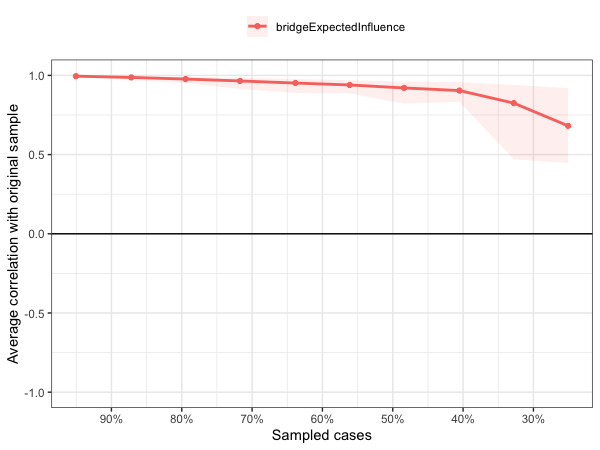
**

**Figure S2:** Case-dropping bootstrapped procedure of Bridge Expected Influence (*model 2)*(number of bootstraps = 1000). The correlations of the centrality measure Bridge Expected Influence between the original sample and those from the subsamples with an increasingly higher percentage of dropout cases were calculated. The correlation stability coefficient (CS coefficient) represents the maximum proportion of cases that can be dropped to retain a correlation of at least 0.70 with the original Bridge Expected Influence in at least 95% of the samples. The 95% confidence interval of the correlation is indicated. The case-dropping bootstrap procedure showed that CS coefficient of node Bridge Expected Influence (CS(cor=0.7) = 0.60) remained high.

**Table S1:** Network analysis BDI-II items and EUROHIS-QOL 8-item index.

| Variable | Expected Influence | Predictability | Edge weight |
| --- | --- | --- | --- |
| b1 | 0.823 | 0.406 | -0.065 |
| b2 | 0.795 | 0.375 | -0.009 |
| b3 | 0.658 | 0.359 | -0.123 |
| b4 | 0.849 | 0.465 | -0.091 |
| b5 | 0.871 | 0.368 | 0.000 |
| b6 | 0.460 | 0.160 | 0.000 |
| b7 | 0.889 | 0.383 | -0.058 |
| b8 | 0.881 | 0.351 | 0.000 |
| b9 | 0.436 | 0.165 | -0.030 |
| b10 | 0.581 | 0.257 | -0.011 |
| b11 | 0.710 | 0.364 | -0.088 |
| b12 | 1.000 | 0.463 | -0.015 |
| b13 | 0.698 | 0.319 | -0.001 |
| b14 | 0.826 | 0.327 | 0.000 |
| b15 | 0.724 | 0.497 | -0.147 |
| b16 | 0.477 | 0.174 | 0.000 |
| b17 | 0.781 | 0.331 | 0.000 |
| b18 | 0.339 | 0.148 | -0.076 |
| b19 | 0.672 | 0.264 | 0.000 |
| b20 | 0.662 | 0.449 | -0.109 |
| b21 | 0.484 | 0.187 | 0.000 |
| QOL8 | -0.958 | 0.428 | / |

Expected Influence in relative values and predictability are given nodewise for the items of the BDI-II (*b1-b21*) and the EUROHIS-QOL 8-item index (*QOL8*). Edge weights refer to the associations between the EUROHIS-QOL 8-item index (*QOL8*) and each of the items of the BDI-II (*b1-b21*). BDI-II: revised Beck Depression Inventory (b1: Sadness; b2: Pessimism; b3: Past failure; b4: Loss of pleasure; b5: Guilty feelings; b6: Punishment feelings; b7: Self-dislike; b8: Self-criticalness; b9: Suicidal thoughts or wishes; b10: Crying; b11: Agitation; b12: Loss of interest; b13: Indecisiveness; b14: Worthlessness; b15: Loss of energy; b16: Changes in sleeping pattern; b17: Irritability; b18: Changes in appetite; b19: Concentration difficulty; b20: Tiredness or fatigue; b21: Loss of interest in sex); EUROHIS-QOL: European Union Health Interview Survey for Quality Of Life.

**Table S2:** Network analysis BDI-II items and EUROHIS-QOL items.

| Variable | Bridge Expected Influence | Edge weight | | | | | | | |
| --- | --- | --- | --- | --- | --- | --- | --- | --- | --- |
|  |  | QOL | HEA | ENE | ACT | YOU | REL | MON | LIV |
| b1 | -0.126 | -0.089 | 0.000 | 0.000 | 0.000 | -0.016 | 0.000 | 0.000 | 0.000 |
| b2 | -0.070 | 0.000 | -0.040 | 0.000 | 0.000 | -0.001 | 0.000 | 0.000 | 0.000 |
| b3 | -0.105 | -0.031 | 0.000 | 0.000 | 0.000 | 0.000 | -0.066 | -0.042 | -0.037 |
| b4 | -0.159 | -0.063 | 0.000 | -0.027 | 0.000 | 0.000 | -0.053 | 0.018 | 0.000 |
| b5 | -0.025 | 0.000 | 0.000 | 0.000 | 0.073 | -0.051 | 0.000 | 0.000 | 0.000 |
| b6 | -0.065 | -0.078 | 0.000 | 0.000 | 0.042 | 0.000 | 0.000 | -0.012 | -0.022 |
| b7 | -0.098 | 0.000 | 0.000 | 0.000 | -0.012 | -0.143 | 0.000 | 0.000 | 0.000 |
| b8 | -0.047 | 0.000 | 0.000 | 0.000 | 0.000 | -0.021 | 0.000 | 0.000 | 0.000 |
| b9 | -0.006 | 0.000 | 0.000 | 0.000 | 0.000 | -0.011 | -0.025 | 0.000 | -0.025 |
| b10 | -0.059 | 0.000 | 0.000 | 0.000 | -0.029 | 0.000 | 0.000 | -0.011 | 0.000 |
| b11 | -0.125 | 0.000 | 0.000 | -0.001 | -0.060 | 0.000 | 0.000 | -0.027 | 0.000 |
| b12 | -0.045 | 0.000 | 0.000 | 0.000 | -0.013 | -0.042 | 0.000 | 0.000 | 0.000 |
| b13 | -0.056 | 0.015 | 0.000 | -0.040 | 0.000 | 0.000 | 0.000 | 0.000 | 0.000 |
| b14 | -0.056 | 0.000 | 0.000 | 0.000 | 0.000 | 0.000 | 0.000 | 0.000 | 0.013 |
| b15 | -0.237 | 0.000 | 0.000 | -0.085 | -0.102 | 0.000 | 0.000 | 0.000 | 0.000 |
| b16 | -0.006 | 0.000 | 0.000 | 0.000 | 0.000 | 0.000 | 0.000 | 0.000 | 0.019 |
| b17 | -0.071 | 0.047 | 0.000 | 0.000 | 0.000 | -0.087 | 0.000 | 0.000 | 0.000 |
| b18 | -0.117 | 0.000 | 0.000 | 0.000 | -0.086 | 0.000 | 0.000 | -0.021 | 0.000 |
| b19 | -0.057 | 0.000 | 0.000 | 0.000 | 0.000 | -0.038 | 0.000 | 0.000 | 0.035 |
| b20 | -0.230 | 0.000 | -0.002 | -0.236 | 0.000 | 0.000 | 0.000 | 0.004 | 0.000 |
| b21 | -0.001 | 0.000 | 0.000 | 0.000 | 0.000 | 0.000 | 0.000 | 0.005 | 0.046 |
| QOL | -0.195 | / | 0.291 | 0.097 | 0.103 | 0.036 | 0.030 | 0.191 | 0.092 |
| HEA | -0.083 | 0.291 | / | 0.136 | 0.186 | 0.107 | 0.000 | 0.000 | 0.000 |
| ENE | -0.393 | 0.097 | 0.136 | / | 0.265 | 0.022 | 0.073 | 0.139 | 0.018 |
| ACT | -0.235 | 0.103 | 0.186 | 0.265 | / | 0.183 | 0.000 | 0.005 | 0.068 |
| YOU | -0.515 | 0.036 | 0.107 | 0.022 | 0.183 | / | 0.391 | 0.000 | 0.000 |
| REL | -0.150 | 0.030 | 0.000 | 0.073 | 0.000 | 0.391 | / | 0.006 | 0.266 |
| MON | -0.167 | 0.191 | 0.000 | 0.139 | 0.005 | 0.000 | 0.006 | / | 0.255 |
| LIV | -0.025 | 0.092 | 0.000 | 0.018 | 0.068 | 0.000 | 0.266 | 0.255 | / |

Bridge Expected Influence is given nodewise for the items of the BDI-II (*b1-b21*) and the eight items of the EUROHIS-QOL in relative values. Edge weights refer to the associations between a particular node and every other node. BDI-II: revised Beck Depression Inventory (b1: Sadness; b2: Pessimism; b3: Past failure; b4: Loss of pleasure; b5: Guilty feelings; b6: Punishment feelings; b7: Self-dislike; b8: Self-criticalness; b9: Suicidal thoughts or wishes; b10: Crying; b11: Agitation; b12: Loss of interest; b13: Indecisiveness; b14: Worthlessness; b15: Loss of energy; b16: Changes in sleeping pattern; b17: Irritability; b18: Changes in appetite; b19: Concentration difficulty; b20: Tiredness or fatigue; b21: Loss of interest in sex); EUROHIS-QOL: European Union Health Interview Survey for Quality Of Life (QOL: How would you rate your quality of life?; HEA: How satisfied are you with your health?; ENE: Do you have enough energy for everyday life?; ACT: How satisfied are you with your ability to perform your daily living activities?; YOU: How satisfied are you with yourself?; REL: How satisfied are you with your personal relationships?; MON: Have you enough money to meet your needs?; LIV: How satisfied are you with the conditions of your living place?).

**Table S3:** Affiliations of the collaborators of the COPPADIS Study Group.

| **Name (Last Name, First Name)** | **Location** | **Role** | **Contribution** |
| --- | --- | --- | --- |
| Astrid Adarmes, Daniela | Hospital Universitario Virgen del Rocío, Sevilla, Spain | Site investigator | Evaluation of participants and/or data management |
| Almeria, Marta | Hospital Universitari Mutua de Terrassa, Terrassa, Barcelona, Spain | Site investigator | Neuropsychologist; evaluation of participants |
| Alonso Losada, Maria Gema | Hospital Álvaro Cunqueiro, Complejo Hospitalario Universitario de Vigo (CHUVI), Vigo, Spain | Site investigator / PI | Coordination at the center  Evaluation of participants and/or data management |
| Alonso Cánovas, Araceli | Hospital Universitario Ramón y Cajal, Madrid, Spain | Site investigator | Evaluation of participants and/or data management |
| Alonso Frech, Fernando | Hospital Universitario Clínico San Carlos, Madrid, Spain | Site investigator | Evaluation of participants and/or data management |
| Alonso Redondo, Ruben | Hospital Universitario Lucus Augusti (HULA), Lugo, Spain | Site investigator / PI | Coordination at the center  Evaluation of participants and/or data management |
| Aneiros Díaz, Ángel | Complejo Hospitalario Universitario de Ferrol (CHUF), Ferrol, A Coruña, Spain | Site investigator / PI | Coordination at the center  Evaluation of participants and/or data management |
| Álvarez, Ignacio | Hospital Universitari Mutua de Terrassa, Terrassa, Barcelona, Spain | Site investigator | Evaluation of participants and/or data management |
| Álvarez Sauco, María | Hospital General Universitario de Elche, Elche, Spain | Site investigator / PI | Coordination at the center  Evaluation of participants and/or data management |
| Arnáiz, Sandra | Complejo Asistencial Universitario de Burgos, Burgos, Spain | Site investigator | Evaluation of participants and/or data management |
| Arribas, Sonia | Hospital Universitari Mutua de Terrassa, Terrassa, Barcelona, Spain | Site investigator | Neuropsychologist; evaluation of participants |
| Ascunce Vidondo, Arancha | Complejo Hospitalario de Navarra, Pamplona, Spain | Site investigator | Evaluation of participants and/or data management |
| Aguilar, Miquel | Hospital Universitari Mutua de Terrassa, Terrassa, Barcelona, Spain | Site investigator | Evaluation of participants and/or data management |
| Ávila Rivera, Maria Asunción | Consorci Sanitari Integral, Hospital General de L´Hospitalet, L´Hospitalet de Llobregat, Barcelona, Spain | Site investigator / PI | Coordination at the center  Evaluation of participants and/or data management |
| Bernardo Lambrich, Noemí | Hospital de Tortosa Verge de la Cinta (HTVC), Tortosa, Tarragona, Spain | Site investigator | Evaluation of participants and/or data management |
| Bejr-Kasem, Helena | Hospital de Sant Pau, Barcelona, Spain | Site investigator | Evaluation of participants and/or data management |
| Blázquez Estrada, Marta | Hospital Universitario Central de Asturias, Oviedo, Spain | Site investigator | Evaluation of participants and/or data management |
| Botí González, Maria Ángeles | Hospital Universitari Mutua de Terrassa, Terrassa, Barcelona, Spain | Site investigator | Neuropsychologist; evaluation of participants |
| Borrué, Carmen | Hospital Infanta Sofía, Madrid, Spain | Site investigator / PI | Coordination at the center  Evaluation of participants and/or data management |
| Buongiorno, Maria Teresa | Hospital Universitari Mutua de Terrassa, Terrassa, Barcelona, Spain | Site investigator | Nurse study coordinator |
| Cabello González, Carolina | Complejo Hospitalario de Navarra, Pamplona, Spain | Site investigator | Scheduling of evaluations |
| Cabo López, Iria | Complejo Hospitalario Universitario de Pontevedra (CHOP), Pontevedra, Spain | Site investigator / PI | Coordination at the center  Evaluation of participants and/or data management |
| Caballol, Nuria | Consorci Sanitari Integral, Hospital Moisés Broggi, Sant Joan Despí, Barcelona, Spain. | Site investigator / PI | Coordination at the center  Evaluation of participants and/or data management |
| Cámara Lorenzo, Ana | Hospital Clínic de Barcelona, Barcelona, Spain | Site investigator | Nurse study coordinator |
| Canfield Medina, Héctor | Complejo Hospitalario Universitario de Ferrol (CHUF), Ferrol, A Coruña, Spain | Site investigator | Evaluation of participants and/or data management |
| Carabajal Pendón, Estefanía | Hospital La Princesa, Madrid, Spain | Site investigator (from MAR/23) | Evaluation of participants and/or data management |
| Carrillo, Fátima | Hospital Universitario Virgen del Rocío, Sevilla, Spain | Site investigator | Evaluation of participants and/or data management |
| Carrillo Padilla, Francisco José | Hospital Universitario de Canarias, San Cristóbal de la Laguna, Santa Cruz de Tenerife, Spain | Site investigator / PI | Coordination at the center  Evaluation of participants and/or data management |
| Casas, Elena | Complejo Asistencial Universitario de Burgos, Burgos, Spain | Site investigator | Evaluation of participants and/or data management |
| Catalán, Maria José | Hospital Universitario Clínico San Carlos, Madrid, Spain | Site investigator / PI | Coordination at the center  Evaluation of participants and/or data management |
| Clavero, Pedro | Complejo Hospitalario de Navarra, Pamplona, Spain | Site investigator | Evaluation of participants and/or data management |
| Cortina Fernández, A | Complejo Hospitalario Universitario de Ferrol (CHUF), Ferrol, A Coruña, Spain | Site investigator | Coordination of blood extractions |
| Cosgaya, Marina | Hospital Clínic de Barcelona, Barcelona, Spain | Site investigator | Evaluation of participants and/or data management |
| Cots Foraster, Anna | Institut d'Assistència Sanitària (IAS) - Instituí Cátala de la Salud. Girona, Spain | Site investigator | Evaluation of participants and/or data management |
| Crespo Cuevas, Ane | Hospital del Mar, Barcelona, Spain. | Site investigator | Evaluation of participants and/or data management |
| Cubo, Esther | Complejo Asistencial Universitario de Burgos, Burgos, Spain | Site investigator / PI | Coordination at the center  Evaluation of participants and/or data management |
| De Deus Fonticoba, Teresa | Complejo Hospitalario Universitario de Ferrol (CHUF), Ferrol, A Coruña, Spain | Site investigator | Nurse study coordinator  Evaluation of participants and/or data management |
| De Fábregues-Boixar, Oriol | Hospital Universitario Vall d´Hebron, Barcelona, Spain | Site investigator / PI | Coordination at the center  Evaluation of participants and/or data management |
| Díez Fairen, M | Hospital Universitari Mutua de Terrassa, Terrassa, Barcelona, Spain | Site investigator | Evaluation of participants and/or data management |
| Dotor García-Soto, Julio | Hospital Universitario Virgen Macarena, Sevilla, Spain | Site investigator / PI | Evaluation of participants and/or data management |
| Erro, Elena | Complejo Hospitalario de Navarra, Pamplona, Spain | Site investigator | Evaluation of participants and/or data management |
| Escalante, Sonia | Hospital de Tortosa Verge de la Cinta (HTVC), Tortosa, Tarragona, Spain | Site investigator / PI | Coordination at the center  Evaluation of participants and/or data management |
| Estelrich Peyret, Elena | Institut d'Assistència Sanitària (IAS) - Instituí Cátala de la Salud. Girona, Spain | Site investigator | Evaluation of participants and/or data management |
| Fernández Guillán, Noelia | Complejo Hospitalario Universitario de Ferrol (CHUF), Ferrol, A Coruña, Spain | Site investigator | Neuroimaging studies |
| Gámez, Pedro | Complejo Asistencial Universitario de Burgos, Burgos, Spain | Site investigator | Evaluation of participants and/or data management |
| Gallego, Mercedes | Hospital La Princesa, Madrid, Spain | Site investigator | Evaluation of participants and/or data management |
| García Caldentey, Juan | Centro Neurológico Oms 42, Palma de Mallorca, Spain | Site investigator / PI | Coordination at the center  Evaluation of participants and/or data management |
| García Campos, Cristina | Hospital Universitario Virgen Macarena, Sevilla, Spain | Site investigator | Evaluation of participants and/or data management |
| García Díez, Cristina | Complejo Hospitalario Universitario de Pontevedra (CHOP), Pontevedra, Spain | Site investigator (from MAY/22) | neuropsychologist; evaluation of participants |
| García Moreno, Jose Manuel | Hospital Universitario Virgen Macarena, Sevilla, Spain | Site investigator / PI (until MAR/21) | Coordination at the center  Evaluation of participants and/or data management |
| Gastón, Itziar | Complejo Hospitalario de Navarra, Pamplona, Spain | Site investigator / PI | Coordination at the center  Evaluation of participants and/or data management |
| Gómez Garre, María del Pilar | Hospital Universitario Virgen del Rocío, Sevilla, Spain | Site investigator | Genetic studies coordination |
| Gómez Mayordomo, Víctor | Hospital Clínico San Carlos, Madrid, Spain | Site investigator* | Evaluation of participants and/or data management |
| González Aloy, Javier | Institut d'Assistència Sanitària (IAS) - Instituí Cátala de la Salud. Girona, Spain | Site investigator | Evaluation of participants and/or data management |
| González Aramburu, Isabel | Hospital Universitario Marqués de Valdecilla, Santander, Spain | Site investigator | Evaluation of participants and/or data management |
| González Ardura, Jessica | Hospital Universitario Lucus Augusti (HULA), Lugo, Spain | Site investigator / PI (until FEB/21) | Evaluation of participants and/or data management |
| González García, Beatriz | Hospital La Princesa, Madrid, Spain | Site investigator | Nurse study coordinator |
| González Palmás, Maria Josefa | Complejo Hospitalario Universitario de Pontevedra (CHOP), Pontevedra, Spain | Site investigator | Evaluation of participants and/or data management |
| González Toledo, Gabriel Ricardo | Hospital Universitario de Canarias, San Cristóbal de la Laguna, Santa Cruz de Tenerife, Spain | Site investigator | Evaluation of participants and/or data management |
| Golpe Díaz, Ana | Complejo Hospitalario Universitario de Ferrol (CHUF), Ferrol, A Coruña, Spain | Site investigator | Laboratory analysis coordination |
| Grau Solá, Mireia | Consorci Sanitari Integral, Hospital Moisés Broggi, Sant Joan Despí, Barcelona, Spain | Site investigator | Evaluation of participants and/or data management |
| Guardia, Gemma | Hospital Universitari Mutua de Terrassa, Terrassa, Barcelona, Spain | Site investigator | Evaluation of participants and/or data management |
| Hernández Vara, Jorge | Hospital Universitario Vall d´Hebron, Barcelona, Spain | Site investigator / PI | Coordination at the center  Evaluation of participants and/or data management |
| Horta Barba, Andrea | Hospital de Sant Pau, Barcelona, Spain | Site investigator | Neuropsychologist; evaluation of participants |
| Idoate Calderón, Daniel | Complejo Hospitalario Universitario de Pontevedra (CHOP), Pontevedra, Spain | Site investigaor (until MAY/22) | neuropsychologist; evaluation of participants |
| Infante, Jon | Hospital Universitario Marqués de Valdecilla, Santander, Spain | Site investigator / PI | Coordination at the center  Evaluation of participants and/or data management |
| Jesús, Silvia | Hospital Universitario Virgen del Rocío, Sevilla, Spain | Site investigator | Evaluation of participants and/or data management |
| Kulisevsky, Jaime | Hospital de Sant Pau, Barcelona, Spain | Site investigator / PI | Coordination at the center  Evaluation of participants and/or data management |
| Kurtis, Mónica | Hospital Ruber Internacional, Madrid, Spain | Site investigator / PI | Coordination at the center  Evaluation of participants and/or data management |
| Labandeira, Carmen | Hospital Álvaro Cunqueiro, Complejo Hospitalario Universitario de Vigo (CHUVI), Vigo, Spain | Site investigator | Evaluation of participants and/or data management |
| Labrador Espinosa, Miguel Ángel | Hospital Universitario Virgen del Rocío, Sevilla, Spain | Site investigator | Neuroimaging data analysis |
| Lacruz, Francisco | Complejo Hospitalario de Navarra, Pamplona, Spain | Site investigator | Evaluation of participants and/or data management |
| Lage Castro, Melva | Complejo Hospitalario Universitario de Pontevedra (CHOP), Pontevedra, Spain | Site investigator | Evaluation of participants and/or data management |
| Lastres Gómez, Sonia | Complejo Hospitalario Universitario de Pontevedra (CHOP), Pontevedra, Spain | Site investigator | Neuropsychologist; evaluation of participants |
| Legarda, Inés | Hospital Universitario Son Espases, Palma de Mallorca, Spain | Site investigator / PI | Coordination at the center  Evaluation of participants and/or data management |
| López Ariztegui, Nuria | Complejo Hospitalario de Toledo, Toledo, Spain | Site investigator / PI | Evaluation of participants and/or data management |
| López Díaz, Luis Manuel | Hospital Da Costa de Burela, Lugo, Spain | Site investigator (until DEC/16) | Evaluation of participants and/or data management |
| López Domínguez, Daniel | Institut d'Assistència Sanitària (IAS) - Instituí Cátala de la Salud. Girona, Spain | Site investigator | Evaluation of participants and/or data management |
| López Manzanares, Lydia | Hospital La Princesa, Madrid, Spain | Site investigator / PI | Coordination at the center  Evaluation of participants and/or data management |
| López Seoane, Balbino | Complejo Hospitalario Universitario de Ferrol (CHUF), Ferrol, A Coruña, Spain | Site investigator | Neuroimaging studies |
| Lucas del Pozo, Sara | Hospital Universitario Vall d´Hebron, Barcelona, Spain | Site investigator | Evaluation of participants and/or data management |
| Macías, Yolanda | Fundación Hospital de Alcorcón, Madrid, Spain | Site investigator | Evaluation of participants and/or data management |
| Mata, Marina | Hospital Infanta Sofía, Madrid, Spain | Site investigator | Evaluation of participants and/or data management |
| Martí Andres, Gloria | Hospital Universitario Vall d´Hebron, Barcelona, Spain | Site investigator | Evaluation of participants and/or data management |
| Martí, Maria José | Hospital Clínic de Barcelona, Barcelona, Spain | Site investigator / PI | Coordination at the center  Evaluation of participants and/or data management |
| Martínez Castrillo, Juan Carlos | Hospital Universitario Ramón y Cajal, Madrid, Spain | Site investigator /PI | Coordination at the center  Evaluation of participants and/or data management |
| Martinez-Martin, Pablo | Centro Nacional de Epidemiología y CIBERNED, Instituto de Salud Carlos III. Madrid | Collaborator in statistical and methods analysis | Methods and statistical reviewer |
| McAfee, Darrian | University of Pennsylvania, Philadelphia | Collaborator in english style | English style reviewer |
| Meitín, Maria Teresa | Hospital Da Costa de Burela, Lugo, Spain | Site investigator | Evaluation of participants and/or data management |
| Menéndez González, Manuel | Hospital Universitario Central de Asturias, Oviedo, Spain | Site investigator / PI | Coordination at the center  Evaluation of participants and/or data management |
| Méndez del Barrio, Carlota | Hospital Universitario Virgen del Rocío, Sevilla, Spain | Site investigator | Evaluation of participants and/or data management |
| Mendoza Plasencia, Zebenzui | Hospital Universitario de Canarias, San Cristóbal de la Laguna, Santa Cruz de Tenerife, Spain | Site investigator | Evaluation of participants and/or data management |
| Mir, Pablo | Hospital Universitario Virgen del Rocío, Sevilla, Spain | Site investigator / PI | Coordination at the center  Evaluation of participants and/or data management |
| Miranda Santiago, Javier | Complejo Asistencial Universitario de Burgos, Burgos, Spain | Site investigator | Evaluation of participants and/or data management |
| Morales Casado, Maria Isabel | Complejo Hospitalario de Toledo, Toledo, Spain. | Site investigator | Evaluation of participants and/or data management |
| Moreno Diéguez, Antonio | Complejo Hospitalario Universitario de Ferrol (CHUF), Ferrol, A Coruña, Spain | Site investigator | Neuroimaging studies |
| Muro García, Inés | Hospital La Princesa, Madrid, Spain | Site investigator (from MAR/23) | Evaluation of participants and/or data management |
| Nogueira, Víctor | Hospital Da Costa de Burela, Lugo, Spain | Site investigator / PI (until 2022)** | Coordination at the center  Evaluation of participants and/or data management |
| Novo Amado, Alba | Complejo Hospitalario Universitario de Ferrol (CHUF), Ferrol, A Coruña, Spain | Site investigator | Neuroimaging studies |
| Novo Ponte, Sabela | Hospital Universitario Puerta de Hierro, Madrid, Spain. | Site investigator | Evaluation of participants and/or data management |
| Ordás, Carlos | Hospital Rey Juan Carlos, Madrid, Spain, Madrid, Spain. | Site Investigator | Evaluation of participants and/or data management |
| Pagonabarraga, Javier | Hospital de Sant Pau, Barcelona, Spain | Site investigator | Evaluation of participants and/or data management |
| Pareés, Isabel | Hospital Ruber Internacional, Madrid, Spain | Site investigator | Evaluation of participants and/or data management |
| Pascual-Sedano, Berta | Hospital de Sant Pau, Barcelona, Spain | Site Investigator | Evaluation of participants and/or data management |
| Pastor, Pau | Hospital Universitari Mutua de Terrassa, Terrassa, Barcelona, Spain | Site investigator | Evaluation of participants and/or data management |
| Pérez Fuertes, Aída | Complejo Hospitalario Universitario de Ferrol (CHUF), Ferrol, A Coruña, Spain | Site investigator | Blood analysis |
| Pérez Noguera, Rafael | Hospital Universitario Virgen Macarena, Sevilla, Spain | Site investigator | Evaluation of participants and/or data management |
| Planas-Ballvé, Ana | Consorci Sanitari Integral, Hospital Moisés Broggi, Sant Joan Despí, Barcelona, Spain | Site investigator | Evaluation of participants and/or data management |
| Planellas, Lluís | Hospital Clínic de Barcelona, Barcelona, Spain | Site investigator (until DEC/19) | Evaluation of participants and/or data management |
| Prats, Marian Ángeles | Institut d'Assistència Sanitària (IAS) - Instituí Cátala de la Salud. Girona, Spain | Site investigator | Evaluation of participants and/or data management |
| Prieto Jurczynska, Cristina | Hospital Rey Juan Carlos, Madrid, Spain, Madrid, Spain | Site investigator / PI | Coordination at the center  Evaluation of participants and/or data management |
| Puente, Víctor | Hospital del Mar, Barcelona, Spain | Site investigator / PI | Coordination at the center  Evaluation of participants and/or data management |
| Pueyo Morlans, Mercedes | Hospital Universitario de Canarias, San Cristóbal de la Laguna, Santa Cruz de Tenerife, Spain | Site investigator | Evaluation of participants and/or data management |
| Puig Daví, Arnau | Hospital de Sant Pau, Barcelona, Spain | Site einvestigator | Evaluation of participants and/or data management |
| Redondo, Nuria | Hospital La Princesa, Madrid, Spain | Site Investigator | Evaluation of participants and/or data management |
| Rodríguez Méndez, Luisa | Complejo Hospitalario Universitario de Ferrol (CHUF), Ferrol, A Coruña, Spain | Site investigator | Blood analysis |
| Rodríguez Pérez, Amparo Belén | Hospital General Universitario de Elche, Elche, Spain | Site investigator | Evaluation of participants and/or data management |
| Roldán, Florinda | Hospital Universitario Virgen del Rocío, Sevilla, Spain | Site investigator | Neuroimaging studies |
| Ruíz de Arcos, María | Hospital Universitario Virgen Macarena, Sevilla, Spain. | Site investigator | Evaluation of participants and/or data management |
| Ruíz Martínez, Javier | Hospital Universitario Donostia, San Sebastián, Spain | Site investigator | Evaluation of participants and/or data management |
| Sánchez Alonso, Pilar | Hospital Universitario Puerta de Hierro, Madrid, Spain | Site investigator | Evaluation of participants and/or data management |
| Sánchez-Carpintero, Macarena | Complejo Hospitalario Universitario de Ferrol (CHUF), Ferrol, A Coruña, Spain | Site investigator | Neuroimaging studies |
| Sánchez Díez, Gema | Hospital Universitario Ramón y Cajal, Madrid, Spain | Site investigator | Evaluation of participants and/or data management |
| Sánchez Rodríguez, Antonio | Hospital Universitario Marqués de Valdecilla, Santander, Spain | Site investigator | Evaluation of participants and/or data management |
| Santacruz, Pilar | Hospital Clínic de Barcelona, Barcelona, Spain | Site investigator | Evaluation of participants and/or data management |
| Santos García, Diego | CHUAC, Complejo Hospitalario Universitario de A Coruña | Coordinator of the Project | Coordination of the COPPADIS-2015 |
| Segundo Rodríguez, José Clemente | Complejo Hospitalario de Toledo, Toledo, Spain | Site investigator | Evaluation of participants and/or data management |
| Seijo, Manuel | Complejo Hospitalario Universitario de Pontevedra (CHOP), Pontevedra, Spain | Site investigator / PI | Coordination at the center  Evaluation of participants and/or data management |
| Sierra, María | Hospital Universitario Marqués de Valdecilla, Santander, Spain | Site investigator | Evaluation of participants and/or data management |
| Solano, Berta | Institut d'Assistència Sanitària (IAS) - Instituí Cátala de la Salud. Girona, Spain | Site investigator / PI | Coordination at the center  Evaluation of participants and/or data management |
| Suárez Castro, Ester | Complejo Hospitalario Universitario de Ferrol (CHUF), Ferrol, A Coruña, Spain | Site investigator | Evaluation of participants and/or data management |
| Tartari, Juan Pablo | Hospital Universitari Mutua de Terrassa, Terrassa, Barcelona, Spain | Site investigator | Evaluation of participants and/or data management |
| Valero, Caridad | Hospital Arnau de Vilanova, Valencia, Spain | Site investigator | Evaluation of participants and/or data management |
| Vargas, Laura | Hospital Universitario Virgen del Rocío, Sevilla, Spain | Site investigator | Evaluation of participants and/or data management |
| Vela, Lydia | Fundación Hospital de Alcorcón, Madrid, Spain | Site investigator / PI | Coordination at the center  Evaluation of participants and/or data management |
| Villanueva, Clara | Hospital Universitario Clínico San Carlos, Madrid, Spain | Site investigator | Evaluation of participants and/or data management |
| Vives, Bárbara | Hospital Universitario Son Espases, Palma de Mallorca, Spain | Site investigator | Evaluation of participants and/or data management |

*Neurology Department, Institute of Neuroscience, Vithas Madrid La Milagrosa University Hospital, Vithas Hospital Group. Madrid, Spain.

**Neurology Department, Hospital Universitario Lucus Augusti, Lugo, Spain.
